# Continuous capnography monitoring during transport of critically ill patients

**DOI:** 10.1186/s13054-016-1260-2

**Published:** 2016-04-07

**Authors:** Kumaresh Venkatesan

**Affiliations:** Anaesthesia and Intensive Care, Khoo Teck Puat Hospital, Singapore, Singapore

Jia et al. [1] should be congratulated for their large prospective multi-centre observational study to identify the incidence and risk factors of adverse events (AEs) during intra-hospital transport (IHT) of critically ill patients. They a priori categorised the AEs associated with the transfer process into equipment related AEs (E-AEs) and those affecting patient stability (P-AEs). About 44 % (196) of the patients were receiving invasive ventilation during IHT. Although the overall incidence of reported AEs is high (79.8 %), the E-AEs (disconnection/depletion of oxygen supply, loss of ventilator power) and P-AEs (accidental extubation, airway obstruction) were reportedly low. However, a significantly higher proportion had arterial blood gas analysis-related P-AEs with abnormal partial pressure of oxygen in arterial blood (PaO_2_) and partial pressure of carbon dioxide in arterial blood (PaCO_2_). Logistic regression analysis indicated that ventilation was not a risk factor for P-AEs, in contrast to the previously published literature.

Unfortunately, none of the patients in the study were monitored with continuous capnography, which has been recommended as a standard of monitoring in mechanically ventilated intensive care unit (ICU) patients during IHT by European, Australasian and American guidelines [2]. Capnography also has the added potential of providing non-invasive measurement of cardiac output, physiological dead space and total CO_2_ production. Continuous capnography monitoring has now been recommended in all mechanically ventilated ICU patients [3].

The lack of continuous capnography monitoring in patients receiving invasive ventilation during IHT introduces a significant bias into the findings of the study by Jia et al.; it either underestimates the E-AEs, by failing to identify true E-AEs, or overestimates the P-AEs, reflected by the reported higher incidence of abnormal blood gas values due to delayed identification of inappropriate or inadequate ventilation-related events. The authors also did not study the influence of accompanying medical personnel (grade, training and experience in IHT) on the incidence of AEs. Hence, the findings of the study should be interpreted with caution.

## Abbreviations

AE: Adverse event
E-AE: Equipment-related adverse events
ICU: Intensive care unit
IHT: Intra-hospital transport
P-AE: Patient-related adverse event

## References

[1] Jia L, Wang H, Gao Y, Liu H, Yu K (2016). High incidence of adverse events during intra-hospital transport of critically ill patients and new related risk factors: a prospective, multicenter study in China. Crit Care..

[2] Fanara B, Manzon C, Barbot O, Desmettre T, Capellier G (2010). Recommendations for the intra-hospital transport of critically ill patients. Crit Care..

[3] Capnography guidelines. http://www.ics.ac.uk/ics-homepage/guidelines-and-standards/

